# Indirect Immunofluorescence Assay for the Simultaneous Detection of Antibodies against Clinically Important Old and New World Hantaviruses

**DOI:** 10.1371/journal.pntd.0002157

**Published:** 2013-04-04

**Authors:** Sabine Lederer, Erik Lattwein, Merle Hanke, Karen Sonnenberg, Winfried Stoecker, Åke Lundkvist, Antti Vaheri, Olli Vapalahti, Paul K. S. Chan, Heinz Feldmann, Daryl Dick, Jonas Schmidt-Chanasit, Paula Padula, Pablo A. Vial, Raluca Panculescu-Gatej, Cornelia Ceianu, Paul Heyman, Tatjana Avšič-Županc, Matthias Niedrig

**Affiliations:** 1 EUROIMMUN Medizinische Labordiagnostika AG, Luebeck, Germany; 2 Swedish Institute for Infectious Disease Control, Karolinska Institute, Stockholm, Sweden; 3 Department of Virology, Haartman Institute, University of Helsinki, Helsinki, Finland; 4 Department of Microbiology, Faculty of Medicine, Chinese University of Hong Kong, Hong Kong, People's Republic of China; 5 Special Pathogens Program, Zoonotic Diseases and Special Pathogens, National Microbiology Laboratory, Public Health Agency of Canada, Canadian Science Centre for Human and Animal Health, Winnipeg, Manitoba, Canada; 6 WHO Collaborating Centre for Arbovirus and Haemorrhagic Fever Reference and Research, Bernhard Nocht Institute for Tropical Medicine, Hamburg, Germany; 7 Departamento de Virología, Instituto Nacional de Enfermedades Infecciosas, A.N.L.I.S. ‘Dr. Carlos G. Malbrán,’ Buenos Aires, Argentina; 8 Clínica Alemana School of Medicine, Institute of Science, Universidad del Desarrollo, Santiago, Chile; 9 Laboratory of Vector-Borne Infections and Medical Entomology, CANTACUZINO National Institute for Research and Development in Microbiology and Immunology, Bucharest, Romania; 10 Research Laboratory for Vector-Borne Diseases, Queen Astrid Military Hospital, Brussels, Belgium; 11 Institute of Microbiology and Immunology, Laboratory for Diagnosis of Zoonoses and WHO Laboratory, Ljubljana, Slovenia; 12 Center for Biological Safety (ZBS-1), Robert Koch Institute, Berlin, Germany; RTI International, United States of America

## Abstract

In order to detect serum antibodies against clinically important Old and New World hantaviruses simultaneously, multiparametric indirect immunofluorescence assays (IFAs) based on biochip mosaics were developed. Each of the mosaic substrates consisted of cells infected with one of the virus types Hantaan (HTNV), Puumala (PUUV), Seoul (SEOV), Saaremaa (SAAV), Dobrava (DOBV), Sin Nombre (SNV) or Andes (ANDV). For assay evaluation, serum IgG and IgM antibodies were analyzed using 184 laboratory-confirmed hantavirus-positive sera collected at six diagnostic centers from patients actively or previously infected with the following hantavirus serotypes: PUUV (Finland, n = 97); SEOV (China, n = 5); DOBV (Romania, n = 7); SNV (Canada, n = 23); ANDV (Argentina and Chile, n = 52). The control panel comprised 89 sera from healthy blood donors. According to the reference tests, all 184 patient samples were seropositive for hantavirus-specific IgG (n = 177; 96%) and/or IgM (n = 131; 72%), while all control samples were tested negative. In the multiparametric IFA applied in this study, 183 (99%) of the patient sera were IgG and 131 (71%) IgM positive (accordance with the reference tests: IgG, 96%; IgM, 93%). Overall IFA sensitivity for combined IgG and IgM analysis amounted to 100% for all serotypes, except for SNV (96%). Of the 89 control sera, 2 (2%) showed IgG reactivity against the HTNV substrate, but not against any other hantavirus. Due to the high cross-reactivity of hantaviral nucleocapsid proteins, endpoint titrations were conducted, allowing serotype determination in >90% of PUUV- and ANDV-infected patients. Thus, multiparametric IFA enables highly sensitive and specific serological diagnosis of hantavirus infections and can be used to differentiate PUUV and ANDV infection from infections with Murinae-borne hantaviruses (e.g. DOBV and SEOV).

## Author Summary

Hantaviruses are the causative agents of hemorrhagic fever with renal syndrome (HFRS) and hantavirus cardiopulmonary syndrome (HCPS) — serious emerging diseases, with case-fatality rates of up to 15% and about 35%, respectively. So far, over 21 human pathogenic serotypes have been described, which are classified into New World (circulating in the Americas) and Old World (Asia and Europe) hantaviruses. The prodromal phase of hantavirus infections — fever, myalgia, headache and gastrointestinal symptoms — is indistinguishable from those of many other viral infections. The cardiopulmonary phase of HFRS and diuretic phase of HFRS mimic the acute respiratory distress syndrome and renal failure, respectively. In this context, clinical diagnosis has to be confirmed by laboratory testing, which is predominantly based on serology. Although there is an increasing awareness of hantaviruses, infections are still underdiagnosed, in part due to a lack of available standardized serological assays. This study evaluated a commercial multiparametric indirect immunofluorescence assay for the simultaneous detection of antibodies against clinically important Old World (Hantaan, Puumala, Seoul, Saaremaa and Dobrava) and New World (Sin Nombre and Andes) hantaviruses. Test performance was found to be comparable to established highly sensitive and specific in-house assays.

## Introduction

Hantaviruses are enveloped and negative-sense single-stranded RNA viruses of the Bunyaviridae family. The hantavirus genome consists of three segments (L, M, and S), coding for the viral RNA polymerase (L protein), glycoproteins (Gn and Gc) and the nucleocapsid (N) protein, respectively [1]–[5]. The majority of hantaviruses are etiologic agents of either hemorrhagic fever with renal syndrome (HFRS) or hantavirus cardiopulmonary syndrome (HCPS). The number of hantavirus infections is increasing, as reflected by a very recent outbreak at Yosemite National Park (USA; June–August 2012), which put an estimated 10,000 persons at risk of infection and caused several fatal cases [6]. Transmission to humans occurs through the respiratory tract by inhalation of dust and aerosols containing virus-contaminated particles shed by persistently infected viral reservoir species (primarily mice, voles and rats).

So far, over 21 human pathogenic hantavirus serotypes have been described [7]–[9], which are classified into New and Old World hantaviruses according to their worldwide distribution and genetic relatedness. New World hantaviruses include, amongst various others, Andes virus (ANDV) [10] and Sin Nombre virus (SNV) [11], the main causative agents of HCPS in South and North America, respectively, with case-fatality rates of about 35%, mainly due to pulmonary complications and cardiogenic shock [12]. Clinically relevant Old World hantaviruses, predominately distributed in the Eastern Hemisphere, include Dobrava (DOBV) [13], Hantaan (HTNV) [14], Puumala (PUUV) [15], Seoul (SEOV) [16] and Saaremaa (SAAV) virus [17], [18]. The mildest form of HFRS, designated nephropathia epidemica, is caused by PUUV and is associated with a mortality rate of less than 0.1%. SAAV also causes fairly mild HFRS, whereas SEOV, DOBV and HTNV cause moderate to severe HFRS with fatality rates of 1–15% [19].

Due to the rather unspecific symptoms such as headache, backache, myalgia, shivering, abdominal pain and nausea in a high proportion of infected patients, hantavirus syndromes are often clinically misdiagnosed as influenza-like infections, renal failure or idiopathic acute respiratory distress. In this context, implementation of at least one laboratory test is mandatory to support clinical diagnosis. Hantaviruses can be detected either directly by virus isolation or reverse transcriptase polymerase chain reaction (RT-PCR)-based amplification of hantaviral RNA or indirectly by serology [20]. With respect to direct detection, it has to be noted that the level of plasma-associated hantaviral RNA rapidly decreases after the onset of initial symptoms and is suggested to be associated with disease severity (highest RNA load in patients with severe/critical disease) [21]–[23]. RT-PCR in peripheral blood mononuclear cells (PBMC) yields a higher sensitivity, and in most ANDV-infected patients it is successful for up to 3 months after hospitalization (P.A. Vial, unpublished results). Because of the short-termed viremia, detection of hantavirus-specific serum antibodies of class IgM and IgG is most reliable and, thus, widely used for confirmation of hantavirus infection.

For the simultaneous detection of specific serum IgG and IgM against the clinically important hantaviruses, multiparametric indirect immunofluorescence assays (IFAs) were developed based on mosaics of biochips coated with hantavirus-infected cells (positive for serotypes HTNV, PUUV, SEOV, SAAV, DOBV, SNV and ANDV). For assay evaluation, IgG and IgM antibodies were determined in serum panels from healthy blood donors (controls) and confirmed hantavirus-infected patients provided by diagnostic laboratories in six different geographic regions. Previous diagnostic data from these laboratories using mainly in-house or sometimes commercial assays served as reference data.

## Materials and Methods

### Ethics statement

Samples tested were derived from already-existing collections at the indicated institutes and were numerically coded, with the identity of the persons available only to clinicians interacting with the patients or blood donors. Furthermore, patient samples used in this study were routine diagnostic samples taken with patient consent and sent to the respective laboratory to be tested for anti-hantavirus antibodies. Stored aliquots of these anonymized samples were used in this study (conducted in the years 2006–2009) for a comparison of diagnostic methodologies with each sample being tested for the same parameter for which it had initially been screened. The results were not accessible to any outside body and were not reported to the donor or the patient. Therefore written informed consent was not required. Samples were included in this study on the basis of a statement from the Central Ethics Committee of Germany on the use of human samples for research studies [24].

### Patients and serum samples

Assay evaluation was based on 5 serum panels comprising 184 samples from patients in the acute or convalescent phase of hantavirus infection or with past hantavirus infection. Diagnosis of infection was based on clinical (HFRS or HCPS) and serological findings. Characteristics of infected patients are summarized in Table 1. Panel V was a subset of the serum panel previously analyzed by Schmidt and colleagues [25], including 23 sera from Argentina (original sample IDs: 1A, 2A to 5A, 6A, 6B, 7B, 8B to 9B, 11 to 16, 18 to 20) and 29 sera from Chile (original sample IDs: 1CH-A to 2CH-B, 3CH-A to 5CH-B, 6CH-A to 11CH, 13CH, 14CH, 16CH to 22CH). These sera were obtained from 38 patients (7 female, 31 male; median age 30 years; age range 8–77 years), including 13 follow-up patients. Data for gender and age of the other individuals included in this study were not available. The control group comprised 89 serum samples from apparently healthy Canadian (n = 25) and German (n = 64) blood donors (Table 1). All sera were stored at −80°C until analysis.

**Table 1:** Serum panels, clinical characteristics and serological assays.

Serum panel^a^	Diagnostic center	No. of samples	Clinical characteristics	Hantavirus serotype	Reference test system	Multiparametric IFA (Biochip mosaic applied; Fig. 1)
I	Department of Virology, Haartman Institute, University of Helsinki (*Finland*)	97	HFRS	Puumala (PUUV)	Anti-PUUV ELISA (IgM)^b^ and Anti-PUUV IFA (IgG)^c^	Hantavirus Mosaic 1
II	Department of Microbiology, Medical Faculty, Chinese University of Hong Kong (*People's Republic of China*)	5	HFRS	Seoul (SEOV)	IFA (IgG and IgM)^b^	Hantavirus Mosaic 1
III	Laboratory of Vector-Borne Infections and Medical Entomology, CANTACUZINO National Institute for Research and Development in Microbiology and Immunology (Bucharest, *Romania*)	7	HFRS	Dobrava (DOBV)	EIA and/or ELISA (IgM)^c^ EIA/ELISA/WB (IgG)^c^	Hantavirus Mosaic 1
IV	National Microbiology Laboratory, Health Canada (Winnipeg, *Canada*)	23	HCPS	Sin Nombre (SNDV)	Anti-BCCV ELISA (IgG and IgM)^b^	Hantavirus Mosaic 1
V	Universidad del Desarrollo (Santiago, *Chile*) and Hantavirus Laboratory, National Institute of Infectious Diseases (Buenos Aires, *Argentina*)	52	HCPS	Andes (ANDV)	Anti-ANDV ELISA (IgG and IgM)^b^	Hantavirus Mosaic 1 and Hantavirus Mosaic 3
Control sera	University Medical Center Schleswig-Holstein (Luebeck, *Germany*)	64	Healthy	-	Anti-Hantavirus Pool 1 and 2 ELISA (IgG and IgM)^c^	Hantavirus Mosaic 1 and Hantavirus Mosaic 3
	National Microbiology Laboratory, Health Canada (Winnipeg, *Canada*)	25	Healthy	-	Anti-BCCV ELISA (IgG and IgM)^b^	Hantavirus Mosaic 1

a Panel III included 7 sera from 3 follow-up patients and from a patient investigated once; one of the follow-up sera was tested only for IgG in the reference test; Panel V included 25 single samples from 13 Argentinean and 12 Chilean patients as well as 27 serial samples from 13 follow-up patients (5 Argentinean and 8 Chilean) and was tested on both Hantavirus Mosaic 1 and 3. Abbreviations: BCCV, Black Creek Canal virus; EIA, enzyme immunoassay; ELISA, enzyme-linked immunosorbent assay; HCPS, hantavirus cardio-pulmonary syndrome; HFRS, hemorrhagic fever with renal syndrome; IFA, indirect immunofluorescence assay; WB, Western blot.

b In-house assay.

c Commercial assay.

### Serological reference assays

Laboratory confirmation of hantavirus infection was performed at the indicated diagnostic centers shown in Table 1. Regarding panel V, laboratory diagnosis had been performed at the WHO Collaborating Centre for Arbovirus and Haemorrhagic Fever Reference and Research (Hamburg, Germany). Reference tests were characterized as follows (Table 1): panel I, IgG IFA based on PUUV-infected Vero E6 cells, IgM μ-capture ELISA based on lysates of Sf9 insect cells expressing recombinant nucleocapsid (rN) protein of PUUV [26]; panel II, IFA based on HTNV- or SEOV-infected Vero E6 cells; panel III, IgM enzyme immunoassay (EIA) based on rN protein (Dobrava-Hantaan IgM EIA from Reagena, Toivala, Finland, and/or Hantavirus IgM EIA from Focus Diagnostics, Cypress, USA) and rN-protein-based IgG EIA and/or IgG ELISA (Hantavirus IgG EIA from Focus and/or Hantavirus IgG ELISA from Progen, Heidelberg, Germany) or IgG Western blot (recomBlot Bunyavirus IgG from Mikrogen, Neuried, Germany); panel IV, IgG ELISA based on lysates from Black Creek Canal virus (BCCV)-infected Vero E6 cells and IgM capture ELISA based on antigen prepared from BCCV-infected Vero E6 cells [27]; panel V, anti-ANDV ELISA based on yeast-expressed rN protein of ANDV [25]. All patient samples were positive for hantavirus-specific IgG and/or IgM in the corresponding reference tests. In panel III, one follow-up serum from a patient with past DOBV infection was previously tested for IgG only, whereas in the present study both IgG and IgM were determined by means of IFA. Reference tests used for examining the control panel were: Anti-Hantavirus Pool 1 “Eurasia” ELISA (IgG, IgM) based on recombinant nucleocapsid antigens of HTNV, DOBV and PUUV; Anti-Hantavirus Pool 2 “America” ELISA (IgG, IgM) based on recombinant nucleocapsid antigens of ANDV and SNV; anti-BCCV ELISA (IgG, IgM) as described above.

### Multiparametric immunofluorescence assays (IFAs)

**Figure 1:**
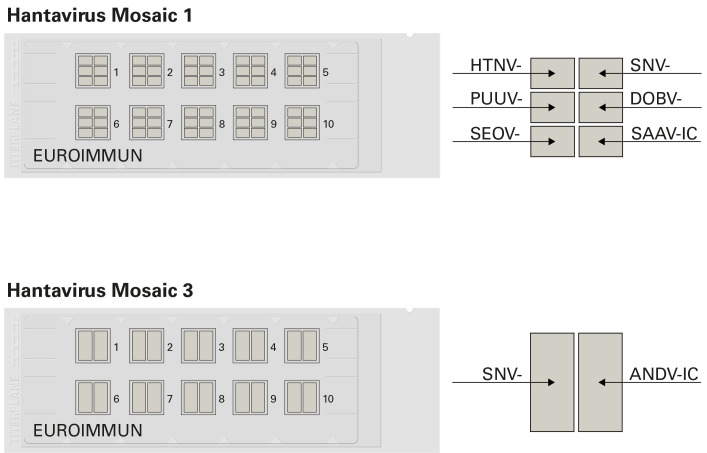
Immunofluorescence microscope slides for the multiparametric detection of hantavirus-specific antibodies. A microscope slide has ten reaction fields, each of which contains a biochip mosaic, allowing ten samples or sample dilutions to be incubated simultaneously with the same range of substrates. Due to identical incubation protocols, IgG and IgM testing can be performed on different reactions fields of the same slide using the respective secondary antibody conjugate. Hantavirus Mosaic 1 comprises mosaics of six biochips coated with Hantaan virus (HTNV)-, Puumala virus (PUUV)-, Seoul virus (SEOV)-, Sin Nombre virus (SNV)-, Dobrava virus (DOBV)- and Saaremaa virus (SAAV)-infected cells (-IC). Hantavirus Mosaic 3 consists of mosaics of two biochips coated with Sin Nombre virus (SNV)- and Andes virus (ANDV)-IC.

For multiparametric IFA-based detection of hantavirus-specific IgG and IgM, the Hantavirus Mosaic 1 and 3 (Euroimmun, Luebeck, Germany; Fig. 1) were used. These assays are CE-marked and validated according to Directive 98/79/EC on in vitro diagnostic medical devices, fulfilling the requirements for standardized and reproducible analyses. The IFAs are based on millimeter-sized fragments of glass slides (biochips) glued side by side on the reaction fields of a microscope slide. Biochips were coated with hantavirus-infected EU14 cells, followed by acetone fixation and gamma irradiation.

For standardized testing, the TITERPLANE Technique (Euroimmun) was applied at room temperature according to the manufacturer's instructions. In brief, serial tenfold dilutions (1∶10 to 1∶10,000) of blinded sera were prepared in sample buffer (Euroimmun). For class IgM antibody determination, serum IgG and rheumatoid factors were first preabsorbed by diluting sera 1∶10 in EUROSORB (Euroimmun), mixing thoroughly and incubating for 15 min. After centrifugation (5 min, 2,000 rpm) the supernatant was diluted serially as described above. Samples were applied to the reaction fields of a reagent tray. Mosaic-containing slides were placed into the corresponding recesses of the reagent tray, where all substrates came into contact with the fluids, and the individual reactions commenced simultaneously. After incubation for 30 min, slides were rinsed with a flush of PBS-Tween (PBS containing 0.2% Tween-20) and immersed in PBS-Tween for 5 min. For detection of bound antibodies, slides were placed on reagent trays prepared with fluorescein isothiocyanate (FITC)-conjugated goat anti-human immunoglobulin. To test for IgG antibodies, the respective reaction fields were loaded with the anti-human-IgG FITC conjugate. Accordingly, the anti-human-IgM FITC conjugate was applied to those reaction fields intended for the detection of IgM. Following a 30-min incubation, slides were washed as described above, embedded with mounting medium, coverslipped and evaluated by fluorescence microscopy. Evaluation was performed independently by at least two experienced laboratory experts without reference to the clinical diagnosis and serological precharacterization data. Positive reactions were characterized by a fine- to coarse-granular immunofluorescence (IF) in the cytoplasm of infected cells. Intensities of specific IF were compared to those of hantavirus-negative and -positive reference sera and scored as negative, weak, moderate or strong. Antibody titers were determined based on the 10-fold dilution series, allowing for assumed intermediate titers (corresponding to a theoretical dilution factor of 3.2). Samples with at least a weak specific IF at a dilution of 1∶100 (cut-off) were considered positive. The reciprocal endpoint titer was defined as the highest sample dilution factor for which a weak specific IF was detected. For example, if a serum showed a strong IF at a dilution of 1∶10 and 1∶100, a moderate IF at 1∶1,000 and a negative IF at 1∶10,000, it was assigned a reciprocal endpoint titer of 3,200. If another serum showed a strong IF at 1∶10 and 1∶100, but only a weak IF at 1∶1,000 and negative IF at 1∶10,000, the reciprocal endpoint titer was 1,000. The groups' reciprocal geometric mean titers (rGMT) were determined using Excel (Microsoft, Redmond, USA). In a group of n samples, the rGMT was calculated as the n^th^ root of the product of the samples' reciprocal endpoint titers.

## Results

### Performance of multiparametric anti-hantavirus IFAs

The overall qualitative performance of multiparametric IFAs in detecting anti-hantavirus antibodies was analyzed by considering those samples as seropositive that showed specific reactivity (cut-off 1∶100) against at least one of the different hantavirus serotypes contained in the biochip mosaics.

As shown in Table 2, the overall agreement between the reference tests and multiparametric IFAs was 96% for IgG and 93% for IgM analysis in the patient sera. Multiparametric IFA-based combined IgG and IgM analyses revealed 100% sensitivity for all serum panels, except for panel IV (96%). With respect to the control cohort, none of the healthy blood donors was antibody positive by the reference tests, whereas 2/89 (2%) were IgG positive for HTNV by IFA (98% specificity).

**Table 2:** Diagnostic performance of the multiparametric anti-hantavirus immunofluorescence assay (IFA).

Serum panel	n	Reference tests			IFA^a^			Discordance IFA versus reference tests		Agreement IFA versus reference tests	
		IgG pos	IgM pos	IgG/IgM pos^b^	IgG pos	IgM pos	IgG/IgM pos^b^	IgG	IgM	IgG	IgM
I-PUUV	97	97 (100%)	52 (54%)	97 (100%)	97 (100%)	54 (56%)	97 (100%)	0 (0%)	4 (4%)	97 (100%)	93 (96%)
II-SEOV	5	5 (100%)	5 (100%)	5 (100%)	5 (100%)	5 (100%)	5 (100%)	0 (0%)	0 (0%)	5 (100%)	5 (100%)
III-DOBV	7^c^	7 (100%)	5 (83%)^c^	7 (100%)	7 (100%)	5 (71%)	7 (100%)	0 (0%)	1 (17%)^c^	7 (100%)	5 (83%)^c^
IV-SNV	23	20 (87%)	17 (74%)	23 (100%)	22 (96%)	19 (83%)	22 (96%)	4 (17%)	4 (17%)	19 (83%)	19 (83%)
V-ANDV	52	48 (92%)	52 (100%)	52 (100%)	52 (100%)	48 (92%)	52 (100%)	4 (8%)	4 (8%)	48 (92%)	48 (92%)
**Panels I–V**	**184** c	**177 (96%)**	**131 (72%)** c	**184 (100%)**	**183 (99%)**	**131 (71%)**	**183 (99%)**	**8 (4%)**	**13 (7%)** c	**176 (96%)**	**170 (93%)** c
Controls	89	0 (0%)	0 (0%)	0 (0%)	2 (2%)	0 (0%)	2 (2%)	2 (2%)	0 (0%)	87 (98%)	89 (100%)

a Positive IFA results are based on serum reactivity (titer ≥1∶100) against at least one of the different hantavirus substrates contained in the biochip mosaics.

b Number of sera tested positive for IgG and/or IgM.

c One of the 7 sera of panel III was tested only for IgG in the reference tests. IgM positive rates (%) determined by the reference tests therefore refer to n = 6 (III-DOBV) and n = 183 (panels I–V).

In detail, among a total of 184 hantavirus-positive sera, 177 (96%) and 183 (99%) were IgG positive in the reference tests and IFA, respectively. All samples of panels I, II and III were anti-hantavirus IgG positive by precharacterization and multiparametric IFA. In panel IV, 20/23 (87%) Canadian HCPS patients had hantavirus-specific IgG according to the ELISA-based precharacterization, while the multiparametric IFA revealed a higher positivity rate of 96% (22/23). Discrepant IgG results were found in 4/23 (17%) samples of this panel, including 3 ELISA IgG negative/IFA IgG positive sera that were obtained during acute SNV infection and were confirmed by RT-PCR (Table 3; sera #CA-22, #CA-23 and #CA-24). Another serum (#CA-12) was ELISA IgG positive/IFA IgG negative. In panel V, the IgG IFA achieved a higher seropositivity rate (52/52, 100%) than the reference test (48/52, 92%). The 4 sera of this panel that were ELISA IgG negative/IFA IgG positive had been drawn during acute infection, i.e. within 3 to 5 days after onset of initial symptoms (Table 3; sera #2B, #13, #18 and #19).

**Table 3:** Serum samples with discordant results between the reference tests and the multiparametric immunofluorescence assay (IFA).

Serum panel	Discordant sera (Sample ID)	Time of sampling (Days after onset)^a^	Reference test (IgG/IgM)	IFA (IgG/IgM)
I–PUUV (n = 97)	FI-21	NA	+/+	+/−
	FI-24	NA	+/−	+/+
	FI-38	NA	+/−	+/+
	FI-55	NA	+/−	+/+
III–DOBV (n = 7)	RO-7	>60d	+/+	+/−
IV–SNV (n = 23)	CA-01	NA	+/+	+/−
	CA-12	NA	+/−	−/−
	CA-15	NA^b^	+/−	+/+
	CA-16	NA	+/−	+/+
	CA-17	NA	+/−	+/+
	CA-22	NA^b^	−/+	+/+
	CA-23	NA^b^	−/+	+/+
	CA-24	NA^b^	−/+	+/+
V–ANDV (n = 52)	2B	3d	−/+	+/+
	13	4d	−/+	+/+
	18	4d	−/+	+/+
	19	5d	−/+	+/+
	3A	213d	+/+	+/−
	6B	31d	+/+	+/−
	1A	395d	+/+	+/−
	3B	335d	+/+	+/−

a Days after onset of initial symptoms; NA, data not available.

b Hantavirus infection was confirmed by Western blot and neutralization test (#CA-15) or by RT-PCR (#CA-22 to #CA-24).

According to the reference tests, 131 (72%) patient sera were IgM positive, referring to a total of 183 sera with IgM precharacterization. Using IFA, 131 (71%) out of all 184 patient samples tested IgM positive. Comparing the performance within each serum panel, IgM positivity rates were equal in panel II (reference test/IFA, 100%/100%), but different in panel I (54%/56%), panel III (83%/71%), panel IV (74%/83%) and panel V (100%/92%). Discordant results were obtained for 13 (7%) of the patient sera, for 7 of which IgM-positivity by precharacterization contrasted with IgM-negativity by IFA. However, 5 of them had been drawn in the convalescent phase of the disease (Table 3; panel III, #RO-7; panel V, #1A, #3A, #3B and #6B). Serum #6B derived from a follow-up patient whose first serum sample (#6A), drawn 16 days after onset of symptoms, was IgM positive in the IFA. The remaining 6 sera with discordant IgM results were negative in the reference tests but positive in the multiparametric IFA (Table 3).

When restricting the evaluation to only the (endemic) serotype-specific substrate, a subset of IFA positive sera (5/184, 3%) was either IgM or IgG negative, indicating the possibility of misdiagnosis when serological screening is limited to the suspected (endemic) serotype. Among these 5 cases, 2 sera from PUUV-infected patients (panel I) showed IgM reactivity on the HTNV substrate only, 2 sera from Canadian SNV-infected patients (panel IV) showed IgG/IgM reactivity on the PUUV substrate only, while the remaining serum from a SEOV-infected Chinese patient (panel II) showed IgM reactivity to HTNV only.

### Multiparametric IFA-based hantavirus serotyping

**Figure 2:**
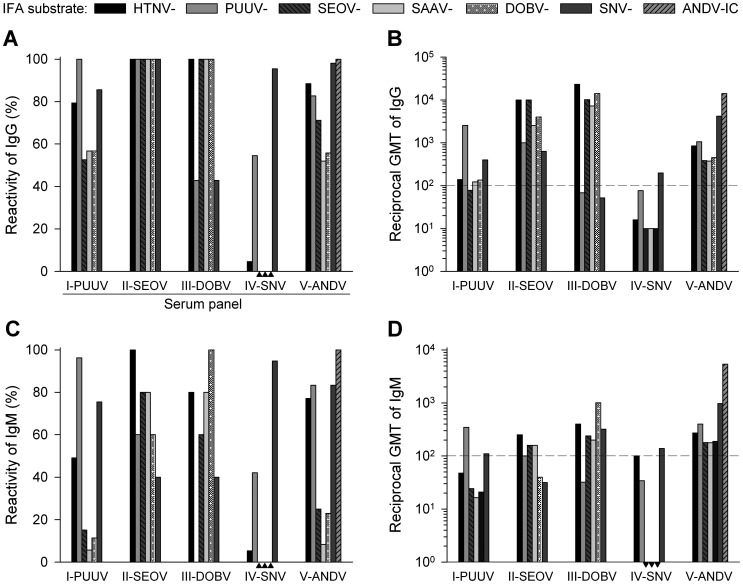
Multiparametric IFA-based reactivity patterns of hantavirus-specific serum antibodies. Bars indicate reactivity rates or reciprocal geometric mean titers (rGMT) for IFA IgG positive sera (**A** and **B**; serum panel: I, n = 97; II, n = 5; III, n = 7; IV, n = 22; V, n = 52) and IFA IgM-positive sera (**C** and **D**; serum panel: I, n = 54; II, n = 5; III, n = 5; IV, n = 19; V, n = 48) for each substrate (cells infected with HTNV, PUUV, SEOV, SAAV, DOBV, SNV and ANDV). Dashed horizontal lines (right panels) indicate the reciprocal cut-off titer; black triangles indicate reactivity rates of 0% (left panels) or rGMTs of <10 (lower right panel). Results for panel IV with ANDV-infected cells were not available.

Depending on the causative hantavirus, cross-reactivity rates of up to 100% were observed when comparing reactivity rates between the seven serotypes used as IFA substrates (Fig. 2 A and C). In general, reciprocal geometric mean titers (rGMTs) of anti-hantavirus IgM were lower than IgG rGMTs (Fig. 2 B and D).

In panel I, all (100%) IFA IgG positive and 96% of the IFA IgM positive sera reacted on PUUV-infected cells (IC), with rGMTs of 2,530 (IgG) and 347 (IgM). Positivity rates of serum IgG/IgM from PUUV-infected patients were also high on SNV-IC (86%/75%) and HTNV-IC (79%/49%), but only moderate (IgG, 53–57%) or low (IgM, 6–15%) on SEOV-, SAAV- and DOBV-IC.

Sera from SEOV-infected patients (panel II) were IgG positive on all tested substrates, while serum IgM revealed highest reactivity (100%) on HTNV-IC. On SEOV- and HTNV-IC, rGMTs of IgG were identical (10,000) or higher compared to those on the other substrates (<4,000); rGMTs of IgM were highest on HTNV-IC (251), followed by SEOV- and SAAV-IC (158).

In accordance with the phylogenetic relatedness of hantaviruses, the highest positivity rates of sera from DOBV-positive patients (panel III) were found on DOBV-, SAAV-, HTNV- and SEOV-IC (IgG/IgM, 100%/60–100%), and markedly lower on PUUV- and SNV-IC (≤43%). The rGMTs of IgG and IgM were highest on HTNV-IC (22,952) and DOBV-IC (1,005), respectively.

Among all hantavirus-infected patients, SNV infections (panel IV) were associated with the lowest serotype-specific rGMTs of 198 (IgG) and 138 (IgM). Serum IgG/IgM from SNV-infected patients reacted on SNV-IC (95%/95%), PUUV-IC (55%/42%) and HTNV-IC (5%/5%), whereas SEOV-, SAAV- and DOBV-IC were negative. These samples were not available for testing on ANDV-IC, but considering the antigenic relatedness of SNV and ANDV, a significant degree of cross-reactivity can be expected, similar to the results obtained for ANDV-infected patients' sera on SNV-IC.

For ANDV-positive sera (panel V), the highest reactivity rates (100%) and rGMTs (IgG/IgM, 13,999/5,389) were detected on ANDV-IC. Slightly less reactivity (98%/83%) and lower rGMTs (1,166/976) were observed on SNV-IC.

**Figure 3:**
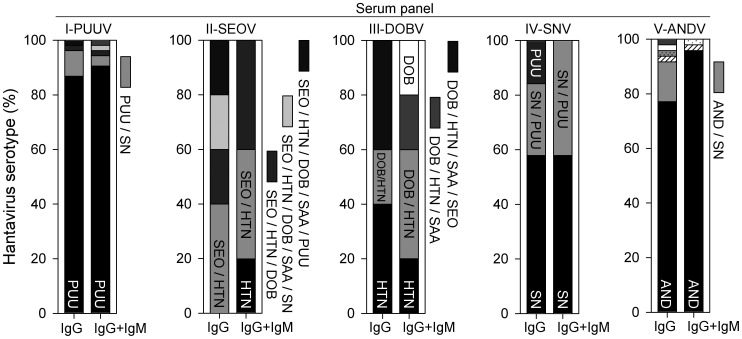
Hantavirus serotype determination by multiparametric indirect immunofluorescence analysis. For sera which were IFA positive for both anti-hantavirus IgG and IgM (serum panel: I, n = 54; II, n = 5; III, n = 5; IV, n = 19; V, n = 48), percentages of hantavirus serotypes with the highest reciprocal IgG endpoint titers were calculated (left bars). Right bars represent results of serotyping by IgG in conjunction with IgM detection. For serum panels I and V, only those serotypes which accounted for more than 2% of the total number of sera are indicated. Results for panel IV with ANDV-infected cells were not available.

Accordingly, serotyping by endpoint titration was successful in the majority of ANDV-infected patients (Fig. 3, panel V), when IgG titers were evaluated separately (77%) or in conjunction with IgM (96%). Regarding panel I, PUUV could be serotyped in 87% and 91% of patients by IgG antibody titration and by combined IgG and IgM analysis, respectively. In panel II and III, a clear serotype could be determined in only a minority of cases due to the high cross-reactivity rates. In panel IV, IgG plus IgM analysis revealed a clear serotype in 58% of the patients; the remaining sera reacted equally on SNV- and PUUV-IC, but SNV could be assigned as the causative agent due to its distribution in North America and the absence of PUUV on the American continent.

## Discussion

Due to the nonspecific clinical symptoms associated with the majority of hantavirus infections, confirmatory laboratory diagnosis is crucial. Generally, acute hantavirus infections are diagnosed serologically by determination of an at least four-fold increase in the IgG titer in consecutive serum samples and/or detection of specific IgM. In-house enzyme-linked immunosorbent assays (ELISAs) and indirect immunofluorescence assays (IFAs) based on antigen from a single or two hantavirus serotypes are widely used for this purpose [28], although these assays may not allow standardized testing of consistently high quality. Furthermore, considering the fact that hantavirus serotypes co-circulate in parts of Europe (PUUV, DOBV and SAAV; [29], [30]), Russia (SAAV, PUUV, DOBV and HTNV; [31]) and Asia (HTNV, SEOV and PUUV; [32], [33]), monospecific tests may fail to detect the causative agent of hantavirus infection, despite the high cross-reactivity rates between closely related hantaviruses [34]. Between the different hantaviruses there are extensive antigenic/serological cross-reactivities that closely follow the phylogenic tree [20]. Thus, the cross-reactivities are especially strong within each group (Murinae-borne, Arvicolinae-borne, Sigmodontinae-borne, Neotominae-borne).

In this study the diagnostic performance of commercial multiparametric hantavirus IFAs based on mosaics of biochips coated with seven different hantavirus-infected cell substrates was evaluated and compared with results from previous laboratory testing using in-house or commercial assays. Among the reference tests were ELISAs using recombinant hantavirus nucleoprotein (rN) as antigen substrate. The N protein represents the major hantavirus antigen and induces an early, strong and long-lasting antibody response [35]–[38]. Recombinant N protein-based assays have been reported to show high sensitivity for hantavirus-specific IgG and IgM [39], [40].

To evaluate the sensitivity and specificity of multiparametric hantavirus IFAs, five laboratory-confirmed hantavirus-positive serum panels obtained from six diagnostic centers located in different geographic regions were used. Multiparametric IFA-based determination of hantavirus-specific IgM and IgG yielded an excellent diagnostic sensitivity of 100% for all panels, except for panel IV (96%). The IFA total seropositivity rate for IgG detection (99%) exceeded that of the reference tests (96%), with an overall agreement of 96%. With respect to the control group, none of the 25 Canadian but 2/64 German healthy blood donors tested IgG positive on the HTNV-IC at the cut-off dilution of 1∶100. These two samples tested negative on the SEOV-, DOBV- and SAAV-IC IFA substrates. Both an anti-hantavirus ELISA and an anti-hantavirus lineblot based on nucleocapsid antigen from PUUV, DOBV, HTNV, SEOV, SNV and ANDV (Euroimmun) were negative, too. Therefore unspecific (false-positive) reactions cannot be ruled out. In the absence of clinical symptoms and without travel history to HTNV endemic regions, such borderline and isolated reactivities on HTNV-IC should be considered as unspecific.

The seropositivity rate of IgM detection by IFA (71%) was almost the same as by the reference methods (72%). However, there was only 93% agreement between the methods, and 13 sera showed discordant IgM results. Among these discordant cases, 7 serum samples had IgM positive reference data, but tested IgM negative by IFA. Five of these sera were drawn in the convalescent phase of a hantavirus infection, namely 1, 7, 11 and 13 months (ANDV-infected patients) and more than 2 months (DOBV-infected patient) after the onset of initial symptoms. Consequently, four of these probably contained persisting IgM antibodies against hantaviral rN protein, since hantavirus-specific IgM usually disappears two to three months after the onset of symptoms [41]. Persistence of IgM antibodies against hantaviral rN protein for as long as two to three years after hospitalization has been reported previously in DOBV-infected patients [42].

With respect to hantavirus-specific IgG, persistence over many years or even life-long may occur, and the IgG response can be delayed in some patients. In the multiparametric IFA, none of the 184 samples was isolated IgM-positive, whereas ELISA-based analysis of sera from SNV-infected patients revealed 3/23 (13%) isolated IgM-positive results. This finding corresponds with recent studies, demonstrating that SNV-specific IgM occurs early after infection, whereas anti-SNV IgG is not detectable in a sizable proportion of sera drawn in the early acute phase [34], [43]. Furthermore, in four sera obtained from ANDV-infected patients 3 to 5 days after onset of initial symptoms, isolated IgM was detected by the ANDV rN protein-based reference ELISA [25]. These discordant IFA/ELISA IgG results could be explained by an earlier appearance of IgG antibodies against hantavirus glycoprotein Gn (formerly termed G1) compared to anti-N protein antibodies as observed previously in acute phase sera [36]. Our data suggest that IgG seroconversion from negative to positive as well as IgM seroconversion from positive to negative is detected earlier by whole native antigen (presented in the IFA) than by recombinant N protein (presented in the ELISA). Considering the need for IgM confirmation by IgG seropositivity, isolated IgM results involve sampling and analysis of at least one consecutive serum sample. Regarding the higher IFA IgG sensitivity, heterogeneous antigen seems to be at least as suited as homogenous antigen to screening for anti-hantavirus IgG in patients suspected of having hantavirus infection.

Notably, multiparametric IFA analysis improved the diagnostic sensitivity, since three samples precharacterized as positive for anti-SEOV IgM and anti-SNV IgM or IgG were found to be only positive on cells infected with closely related hantaviruses (HTNV and PUUV, respectively). This reflects the cross-reacting ability of hantavirus-specific antibodies, which is particularly strong for antibodies against the highly conserved N protein [44]–[49]. As a consequence, serological identification of the causative hantavirus in areas with co-existing serotypes is difficult. Reliable serotyping is particularly important, because severity of syndromes depends on the causative hantavirus serotype [9]. The gold standard for hantavirus serotyping is the neutralization test [50]–[52], which is most reliable but laborious, time-consuming and expensive and has to be performed in a containment laboratory (BSL-3). Serotyping ELISA based on truncated N-proteins have been developed [53]–[55], but can be used as second line diagnostics only, due to a reduced sensitivity. In our study, serotyping by IgG in conjunction with IgM IFA analysis was successful in the majority of HCPS patients infected with ANDV (96%) and HFRS patients infected with PUUV (91%), representing a fast and simple alternative to more elaborate methods. Serotyping failed in patients infected with murinae-borne Old World hantaviruses (DOBV and SEOV), because of their close phylogenetic relatedness with HTNV and SAAV: DOBV N protein has an amino acid sequence identity of 99%, 83% and 80% with the N protein of SAAV, HTNV and SEOV, respectively. Here only neutralization tests or serotyping ELISA can reliably distinguish antibodies raised against these serotypes. However, with respect to the different geographical distribution of HTNV (predominantly South Korea/China/Russia) and DOBV (Balkans) [7], multiparametric IFA-based serotyping in combination with the patient's travel history and clinical characteristics is possible in many cases. For example, in the Romanian hantavirus-infected patients with severe HFRS and without travel history, an infection with HTNV could be excluded, revealing DOBV or SAAV as the causative agent. Infection with SAAV, circulating in Estonia, Finland, Germany, Hungary, Lithuania, Russia, Slovenia and Slovakia [56], could be further excluded because it is associated with milder symptoms.

The worldwide increasing number of hantavirus infection demonstrates the need for reliable serological tests which are simple to perform and allow detection of all clinically relevant hantaviruses. Many hantavirus-infected patients are still misdiagnosed [57]–[61], often due to the lack of generally available standardized assays and of epidemiological data. In line with the most recent European external quality assurance study for hantavirus diagnosis [62], the present study revealed similar performance of IFA and ELISA/EIA. In contrast to the homogenous antigen-presenting ELISA/EIA, the mosaic-based IFA evaluated in this study provides multiparametric testing by combining different substrates of cells infected with clinically relevant Old and New World hantaviruses. Furthermore, unlike in-house IFAs, this commercial assay does not depend on cell culture (establishment of infected cells) and propagation of hantaviruses, since large batches of identical infected cells were created and stored in liquid nitrogen, allowing standardization of immunological analyses. This makes hantavirus testing more widely available to all laboratories familiar with IFA-based diagnostics. In conclusion, analysis of hantavirus-specific IgG and IgM by indirect immunofluorescence on substrate mosaics consisting of cells infected with different hantaviruses is a globally applicable and reliable diagnostic tool for screening of patients suspected of having hantavirus infection, and can be useful for serotyping in areas where hantaviruses of different serogroups are endemic.

## Supporting Information

Figure S1 **STARD flow diagram.** The diagram indicates serum panels from patients and healthy individuals recruited for this study, and the order of serological test execution.
(PDF)

Table S1 STARD table checklist.
(PDF)
