# Correction: Indirect Immunofluorescence Assay for the Simultaneous Detection of Antibodies against Clinically Important Old and New World Hantaviruses

**DOI:** 10.1371/journal.pntd.0008864

**Published:** 2020-11-09

**Authors:** Sabine Lederer, Erik Lattwein, Merle Hanke, Karen Sonnenberg, Winfried Stoecker, Åke Lundkvist, Antti Vaheri, Olli Vapalahti, Paul K. S. Chan, Heinz Feldmann, Daryl Dick, Jonas Schmidt-Chanasit, Paula Padula, Pablo A. Vial, Raluca Panculescu-Gatej, Cornelia Ceianu, Paul Heyman, Tatjana Avšič-Županc, Matthias Niedrig

## Notice of Republication

Following publication of this article [1], concerns were raised about regions of similarity between the images of the indirect immunofluorescence assay (IFA) microscope slides for detection of hantavirus-specific antibodies shown in Fig 1 and images of IFA slides for detection of different antibodies published in other articles [2–4]. The authors clarify that the images in Fig 1 were digitally created using image editing software to combine images of a slide background, reaction fields and assay labels. The images in Fig 1 are for illustrative purposes only, to support understanding of slide configuration and methodology. Fig 1 does not contribute to the results data or scientific conclusions of the study.

In light of concerns that the originally published Fig 1 includes previously published image elements that are not licensed for reproduction and distribution under the terms of the CC-BY license, the original article was republished on October 22, 2020, with a revised Fig 1 in which slide configuration is shown as a schematic diagram. Please download this article again to view the correct version. The revised Fig 1 is provided here for reference.

**Fig 1:**
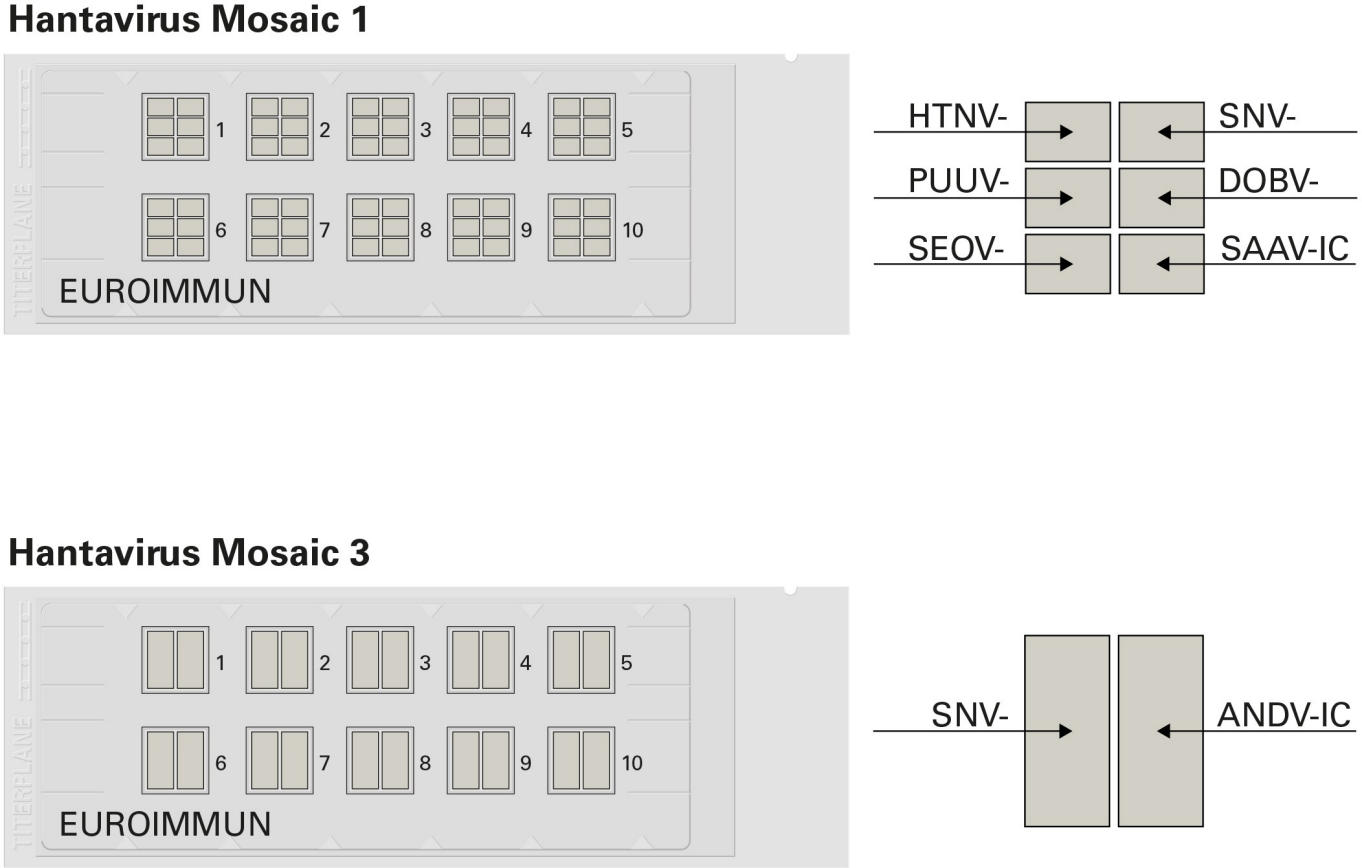
Immunofluorescence microscope slides for the multiparametric detection of hantavirus-specific antibodies. A microscope slide has ten reaction fields, each of which contains a biochip mosaic, allowing ten samples or sample dilutions to be incubated simultaneously with the same range of substrates. Due to identical incubation protocols, IgG and IgM testing can be performed on different reactions fields of the same slide using the respective secondary antibody conjugate. Hantavirus Mosaic 1 comprises mosaics of six biochips coated with Hantaan virus (HTNV)-, Puumala virus (PUUV)-, Seoul virus (SEOV)-, Sin Nombre virus (SNV)-, Dobrava virus (DOBV)- and Saaremaa virus (SAAV)-infected cells (-IC). Hantavirus Mosaic 3 consists of mosaics of two biochips coated with Sin Nombre virus (SNV)- and Andes virus (ANDV)-IC.

The underlying data for this article is available upon request from the corresponding author.
