# Supplementary figures and images for: Indirect Immunofluorescence Assay for the Simultaneous Detection of Antibodies against Clinically Important Old and New World Hantaviruses
Source: PLoS Negl Trop Dis. 2013 Apr 4;7(4):e2157. doi: 10.1371/journal.pntd.0002157 (PMC3617148; doi:10.1371/journal.pntd.0002157)

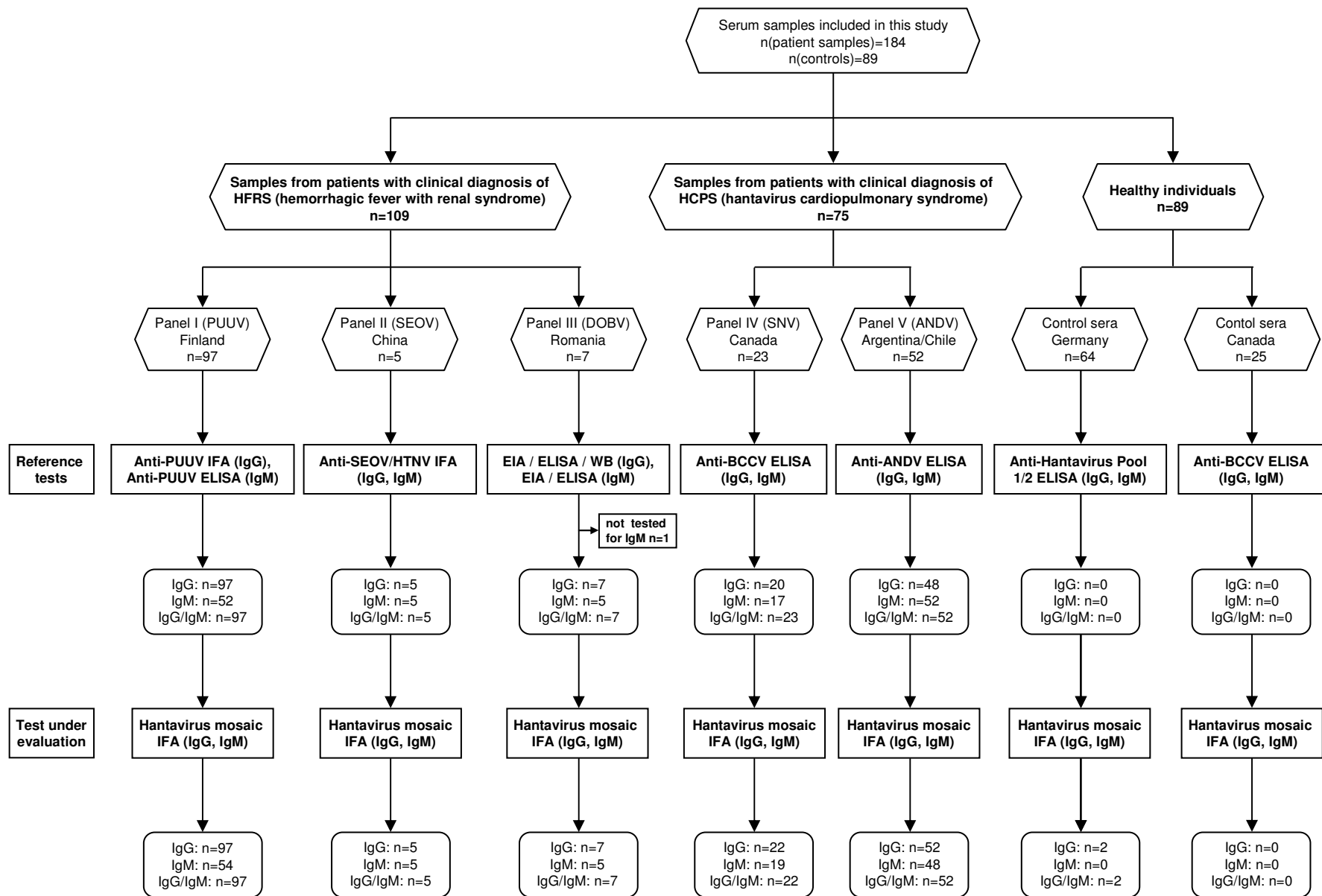

Supplement: Figure S1 — STARD flow diagram. The diagram indicates serum panels from patients and healthy individuals recruited for this study, and the order of serological test execution. (PDF) [file pntd.0002157.s001.pdf]
